# Epistasis and background dependence in the evolution of Omicron variants of the SARS-CoV-2 spike protein

**DOI:** 10.1093/molbev/msag100

**Published:** 2026-05-16

**Authors:** Alief Moulana, Thomas Dupic, Michael M Desai

**Affiliations:** Department of Organismic and Evolutionary Biology, Harvard University, Cambridge, MA, USA; Department of Organismic and Evolutionary Biology, Harvard University, Cambridge, MA, USA; Centre d'Immunologie de Marseille-Luminy, Aix-Marseille University, Marseille, France; Department of Organismic and Evolutionary Biology, Harvard University, Cambridge, MA, USA

**Keywords:** viral evolution, epistasis, host–pathogen coevolution, SARS-CoV-2 spike

## Abstract

The rapid and repeated emergence of severe acute respiratory syndrome coronavirus 2 (SARS-CoV-2) variants, particularly within the Omicron lineage, highlights the virus's remarkable ability to adapt under shifting immune pressures. A central molecular battleground in this evolutionary arms race is the spike receptor-binding domain, which must simultaneously maintain high affinity for the human ACE2 receptor while evading recognition by neutralizing antibodies. In this study, we construct and analyze multiple combinatorial libraries of SARS-CoV-2 receptor-binding domain variants spanning major branches of Omicron evolution, including BA.1, BA.2, BA.5, XBB, and JN.1. Using high-throughput yeast display and binding assays, we map the effects of thousands of mutations and their combinations on ACE2 binding and antibody evasion. Our results reveal that while many receptor-binding domain mutations exhibit additive effects, several mutations interact epistatically in a background-dependent manner. In particular, we identify synergistic interactions between BA.1 and BA.5 mutations that enhance antibody evasion, likely facilitating the rise of recombinant variants and convergent evolution. Conversely, some mutations show lineage-restricted compatibility, suggesting potential constraints on future evolutionary trajectories. Our comprehensive genotype-to-phenotype maps uncover both rugged and smooth regions of the viral fitness landscape and underscore the importance of epistasis in shaping SARS-CoV-2 evolution. These findings improve our ability to anticipate future viral variants and provide a framework for understanding how host–pathogen coevolution unfolds at the molecular level.

## Introduction

The SARS-CoV-2 pandemic has disrupted lives worldwide and placed immense pressure on global healthcare systems, but it has also presented a rare opportunity to observe viral evolution in real time. As the pandemic progressed, the virus continuously evolved in response to shifting population immunity, shaped by vaccination efforts, waves of infection, and public health measures (Markov et al. 2023; Uraki et al. 2026). This ongoing interaction between the virus and host immunity is a prime example of an evolutionary arms race, most evident at the molecular level, where viral antigens and host cell proteins interact.

For SARS-CoV-2, the receptor-binding domain (RBD) of the spike protein plays a central role in this molecular recognition, mediating viral entry through the host ACE2 receptor (Hoffmann et al. 2020; Verdecchia et al. 2020; Walls et al. 2020). At the same time, the host's adaptive immune system mounts a response, primarily through the production of antibodies (Sokal et al. 2021), which compete for binding to the RBD. This puts the RBD under at least two opposing selective pressures: maintaining high affinity for ACE2 while evading recognition by the immune system. As the virus adapts, each new mutation moves the RBD across a fitness landscape (i.e. a map from amino acid sequences to fitness-relevant phenotypes such as antibody evasion and receptor binding). The effects of mutations on these phenotypes determine the virus's ability to persist within individual hosts and spread through the broader population (Bloom and Neher 2023).

The initial phase of the pandemic saw the virus rapidly adapting, leading to several common mutations in the RBD. Some mutations (e.g. N501Y) improved binding affinity to ACE2, while others (e.g. K417N and E484K) helped evade antibodies, giving rise to numerous variants of concern (VOCs) (Harvey et al. 2021; Ramanathan et al. 2021; Zhou et al. 2021). By late 2021, as the Delta wave subsided, the Omicron BA.1 variant emerged. Although genetically very distinct, Omicron was more closely related to an earlier lineage near the ancestral Wuhan-Hu-1 strain than to the then-widespread Delta variant (Sun et al. 2022; Dhama et al. 2023). Compared with Wuhan Hu-1, BA.1 harbors dozens of mutations across its genome, including 31 mutations on the spike protein and 15 within the RBD alone. These unusually high mutation counts allowed BA.1 to evade immunity from prior infections (including Delta and other VOCs) as well as vaccination (Bloom and Neher 2023). As such, BA.1 was hypothesized to have arisen during a prolonged chronic infection, where sustained immune pressure could drive extensive evolution before the variant spread widely.

In previous work, we systematically characterized how combinations of the 15 RBD mutations in Omicron BA.1 affect ACE2 affinity and antibody evasion (Moulana et al. 2022, 2023). High-throughput affinity measurements allowed us to estimate the effects of these mutations both individually and in combination (Adams et al. 2016; Phillips et al. 2021). We demonstrated that, for immune evasion, one or two escape mutations are generally enough to reduce affinity to distinct classes of neutralizing antibodies, with minimal or no interactions between these mutations. In contrast, mutational effects on ACE2 affinity exhibit pervasive epistasis: While individual escape mutations typically reduce ACE2 binding, these deleterious effects are epistatically compensated by the combination of N501Y and Q498R. This gave the BA.1 variant a substantial fitness advantage over Delta and other pre-Omicron strains, allowing it to spread before being subsequently replaced by the BA.2, BA.5, and recombinant XBB subvariants over the next year of its evolution.

Additional mutations on the Omicron background led to varying immune evasion profiles for each subvariant (Cao et al. 2022b; Uraki et al. 2026). First, the BA.2 subvariant, which differs in six RBD residues from BA.1, quickly replaced BA.1 before substantial population immunity to BA.1 was established. Compared with BA.1, BA.2 shows stronger escape from Class 4 antibodies (Iketani et al. 2022), but monoclonal antibodies (mAbs) that BA.1 largely escaped (including Class 3 REGN10987 and COV2-2130) retain neutralizing activity against BA.2 (Bruel et al. 2022; Iketani et al. 2022). BA.2 then diversified into numerous subclades with partially distinct escape profiles, raising the possibility that combining mutations across descendant clades could produce broader evasion (Gruell et al. 2022; Wang et al. 2022; Cao et al. 2023). This potential was realized with the emergence of XBB recombinants, which escaped antibodies that remained effective against earlier Omicron variants (Cao et al. 2023; Wang et al. 2023). However, it remains unclear how broadly these lineage-specific mutations are compatible with each other and what the implications for immune evasion are. Further, which selective pressures lead to which diversified branch of the lineage? Could mutations reappear in a totally different branch, and how likely is recombination between branches—do across-branch mutations interact and, if so, how? More broadly, how rugged is the ACE2 landscape and how do the immune landscapes compare?

As this Omicron lineage diversified and novel mutations arose, by the end of 2023, a new saltation lineage BA.2.86, especially the subvariant JN.1, completely replaced the other subvariants in a rather similar fashion to its BA.1 ancestor (Khan et al. 2023; Yang et al. 2024). Like BA.1, the BA.2.86 lineage emerged from a lineage distantly related to the then-dominant XBB lineage, having accumulated at least ten additional RBD mutations (comparable with the divergence between BA.1 and Wuhan Hu-1 RBD sequences) and thereby evaded broader immunity (Khan et al. 2023; Taylor and Starr 2024; Jian et al. 2025). However, it is unclear whether JN.1 evolution involves a similar epistatic landscape. Some RBD mutations known to enhance viral fitness have appeared on branches that are distant in time (Martin et al. 2021), and previous predictions can sometimes identify the next genotype of interest (Dadonaite et al. 2024). But because of potential epistatic effects, our ability to predict phenotypic effects of specific mutations becomes increasingly limited in more distant genetic backgrounds. It is therefore important to assess how widespread these interactions are across the vast RBD genotypic space.

**Figure 1:**
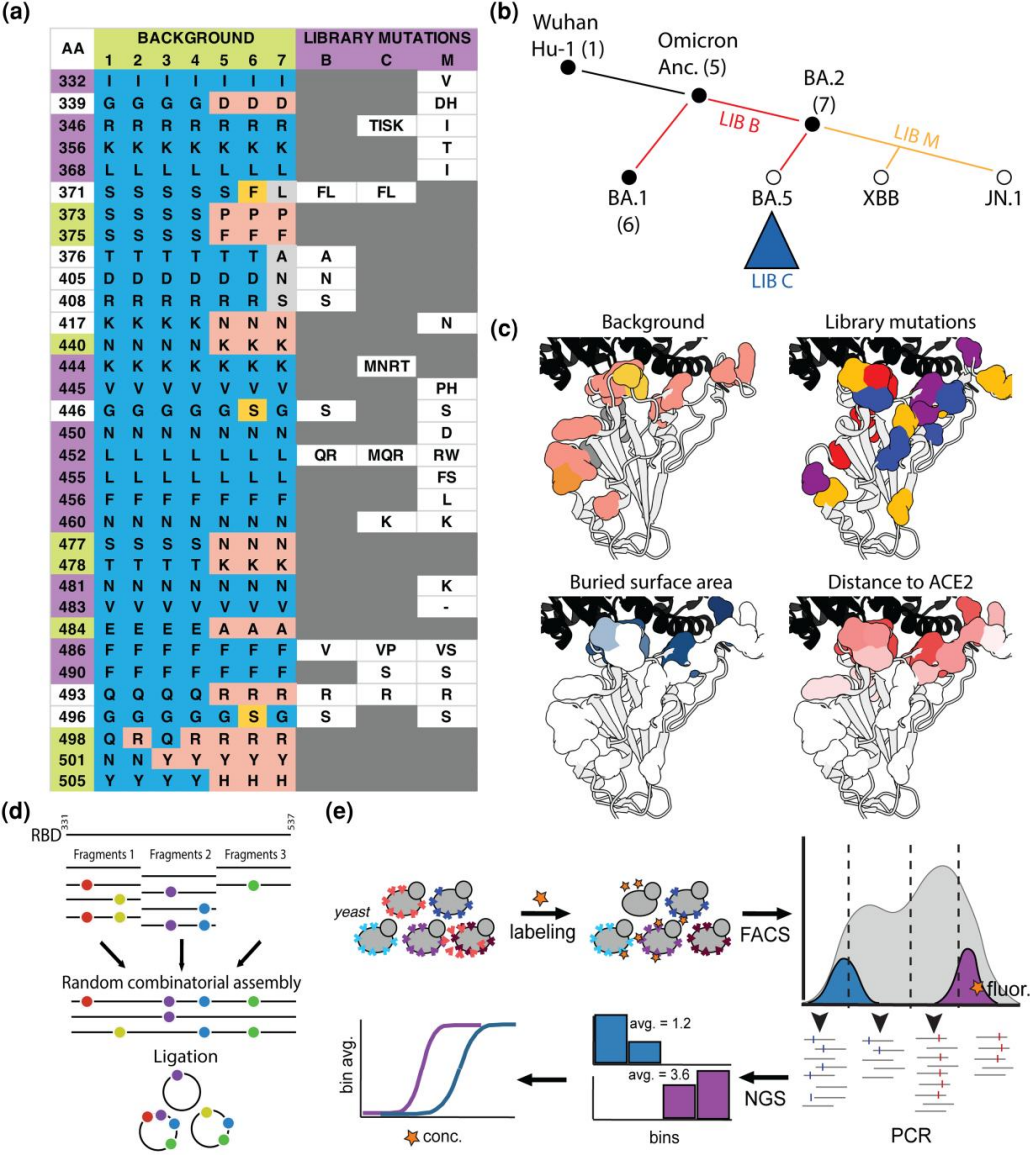
Design, construction, and measurement of combinatorial RBD variant libraries. a) Table summarizing the amino acid positions (*y*-axis) that are mutated in the chosen genetic backgrounds (left: 1 to 7) and in the variant libraries (right: B, C, M). In the leftmost column, positions are highlighted green for background mutations, purple for library mutations, and white for positions mutated in both. The remaining RBD positions are identical to the Wuhan Hu-1 allele. For background mutations, each cell contains the amino acid present at that position in the indicated background (1 to 7). Blue indicates the Wuhan Hu-1 amino acid, pink indicates mutations shared between BA.1 and BA.2 (“Omicron ancestor”), gold indicates BA.1-specific mutations, and silver indicates BA.2-specific mutations. For library mutations, each cell lists the amino acid(s) introduced at that position in the corresponding library. b) Evolutionary tree (black branches) depicting RBD genotypic relationships among selected VOCs (nodes). Colored branches indicate the lineage segments targeted by each library (Library B, red; Library M, orange; Library C indicated by the blue triangle). Backgrounds used as templates for library construction are numbered to correspond to the columns in a). Backgrounds 2 to 4 (not shown) are derived from Background 1 by combinatorial substitutions at residues 498 and 501. c) ACE2/Omicron BA.1 RBD cocrystal structure (PDB: 7WHH; Lan et al. 2022) with ACE2 in black and the RBD in white, with surfaces shown for the positions included in a). Residues are colored by (i) background-mutation class (light red: mutated to the same amino acid in both BA.1 and BA.2; gold: BA.1-specific; silver: BA.2-specific; dark orange: different amino acids across backgrounds), (ii) library membership (red: diversified in Library B only; blue: diversified in Library C only; purple: diversified in both Libraries B and C; orange: diversified in Library M only; Library M sites that overlap with B and/or C are colored according to the corresponding B/C category rather than orange), (iii) buried surface area (light blue → dark blue: small → large), and (iv) shortest distance to ACE2 (light red → dark red: far → near). d) Summary of combinatorial fragment assembly. e) Schematic of library transformation into yeast and the subsequent phenotype measurement workflow (see Materials and methods).

Here, we explore these questions by generating more comprehensive phenotypic measurements across diverse RBD genetic backgrounds. We constructed several spike protein RBD libraries and used a yeast display system to measure binding affinities of each variant to the cognate human ACE2 receptor and to a set of monoclonal antibodies. These RBD variant libraries include but are not limited to variants from BA.1 to JN.1. Specifically, we built four variant libraries: (i) all combinations of ACE2 Omicron BA.1 mutations on the Wuhan Hu-1 background (previously published (Moulana et al. 2022, 2023); (ii) the entire mutational landscape of BA.1, BA.2, BA.4, and BA.5 on a genetic background containing mutations shared by BA.1 and BA.2 (here termed “Omicron ancestor”); (iii) combinations of prevalent mutations in the BA.5 clade (here termed “BA.5C mutations”) on seven divergent genetic backgrounds; and (iv) combinations of XBB.1 and JN.1 mutations on four different genetic backgrounds (Fig. 1a). All these libraries are generated combinatorically, ensuring the appearance of each mutation at least 20 times across the library. These data provide a basis for improving our ability to characterize fitness-relevant genotype-to-phenotype maps and thus to understand and predict evolution in this system.

## Results

### Library choice and generation

In this study, we constructed three yeast display RBD variant libraries and analyzed them alongside the library we previously constructed in Moulana et al. (2022). Each of these libraries was designed to address a different aspect of RBD evolution during the pandemic (Fig. 1b):

- Library A is the original combinatorial library containing all possible combinations of the 15 spike protein RBD mutations that separate Wuhan Hu-1 and Omicron BA.1. The library contains 2^15^ = 32,768 variants. This dataset was previously generated in Moulana et al. (2022) and is included here only for reference where needed.
- Library B spans the entire mutational landscape of BA.1, BA.2, BA.5, and BA.2.12.1, introduced on the Omicron ancestor background. This involves seven biallelic and two triallelic loci, for a total of 2^7^ × 3^2^ = 1,152 variants. This library focuses on studying how mutations, both within and across lineages, interact to affect ACE2 and antibody-binding affinities (Fig. 1d).
- Library C extends our study to include BA.5C mutations introduced in all possible combinations on each of 7 genetic backgrounds (see Fig. 1b), for a total of 7 (backgrounds) × 2^3^ × 3^1^ × 4^1^ × 5^2^ = 16,800 variants. This library investigates how these BA.5C mutations affect ACE2 and antibody affinities across the possible historic backgrounds.
- Library M, assembled combinatorically from synthesized DNA fragments, contains one of four possible genetic backgrounds (Wuhan Hu-1, Omicron ancestor, BA.1, and BA.2). On each background, combinations of 26 mutations common to the new JN.1 and XBB.1 lineages are introduced. This theoretically results in 4 × 2^16^ × 3^5^ = 63,700,920 variants, of which we randomly produced and assayed ∼300,000.

For each library, variant mutations are introduced across multiple genetic backgrounds containing background-specific mutations. Both background and library variant mutations are distributed across the RBD, with a significant proportion clustered around the ACE2-binding motif (Fig. 1c). Mutations on the Omicron ancestor, for instance, are often found at residues in the buried surface area near the ACE2 interface, whereas BA.2 background mutations are generally far from ACE2 contact. Each library was constructed by combinatorially assembling three to four fragments of the RBD sequence. Each fragment has multiple possible variants (all possible combinations of the mutations within that fragment), and during Golden Gate assembly, one version of each fragment is randomly incorporated to produce a single complete RBD sequence (Fig. 1d). Fragments for Libraries B and C were generated by polymerase chain reaction (PCR) using primers with mutation-containing overhangs. Fragments for Library M were synthesized as an oligo pool (Twist Biosciences).

After constructing each library, we performed various binding assays (Fig. 1e; Table 1). These assays broadly measure one of three types of observed phenotypes: absolute binding affinity (*K*_D_), binding fluorescence at one or two ligand concentrations, and enrichment of fluorescence-labeled binders at a single concentration (see Materials and methods for details). The specific ligand and concentration used for each measurement vary depending on the library (Table 1).

**Table 1:** Library assays.

Library	ACE2 assay	mAb assay	List of mAbs
B	Tite-Seq	Tite-Seq	AZD1061, AZD8895, REGN10987
C	Tite-Seq	Sort-Seq (×2)	AZD1061, AZD8895, REGN10987, LY-CoV1404, S309
M	Sort-Seq	Enrichment	AZD1061, AZD8895, REGN10987, LY-CoV1404, S309, 002-S21F2, Iv0221, Iv0262

Overview of the high-throughput measurements (ACE2 and mAb) we performed on each library.

Across the three libraries, we measured affinities to various antibodies, selected for (i) representing an epitope class, (ii) prior evidence of a clear range of evasion across variants included in the library, and (iii) commercial availability. For all three libraries, we measured affinities to the Class 1 antibody AZD8895 and the Class 3 antibodies AZD1061 and REGN10987. These two classes target distinct RBD epitopes (Barnes et al. 2020; Chen et al. 2023) and differ in their efficacy against the two main Omicron clades, BA.1 (still susceptible to class 1) and BA.2 (still susceptible to class 3) (Bruel et al. 2022; Cao et al. 2022b). For Library C, we additionally measured binding to LY-CoV1404, a Class 3 antibody that retains binding against most BA.2 subvariants, including BA.5, but is evaded by some common BA.5 mutations (Hentzien et al. 2022; Liew et al. 2023). For Library M, we further included three Class 3 antibodies (S309, 002-S21F2, and Iv0221), which still bind some of the later BA.2 subvariants (Kumar et al. 2022; Sun et al. 2023). Despite this diverse antibody panel, our measurements are far from capturing the full epitope space and therefore cannot fully represent the immune pressures acting during viral evolution. Nevertheless, they allow us to characterize general structures of the antibody affinity landscapes themselves, especially as spanned by evolution.

Both ACE2 and antibody binding datasets demonstrate strong reproducibility across libraries and assay types. The *K*_D_ measurements for Library B are strongly correlated between replicates (Fig. S1a; *R*^2^ > 0.91). Similarly, fluorescence and enrichment measurements in subsequent libraries also exhibit high correlations (Fig. S1b and c; *R*^2^ > 0.88 and *R*^2^ > 0.76, respectively).

### Beneficial combinations of lineage-specific mutations (Library B)

**Figure 2:**
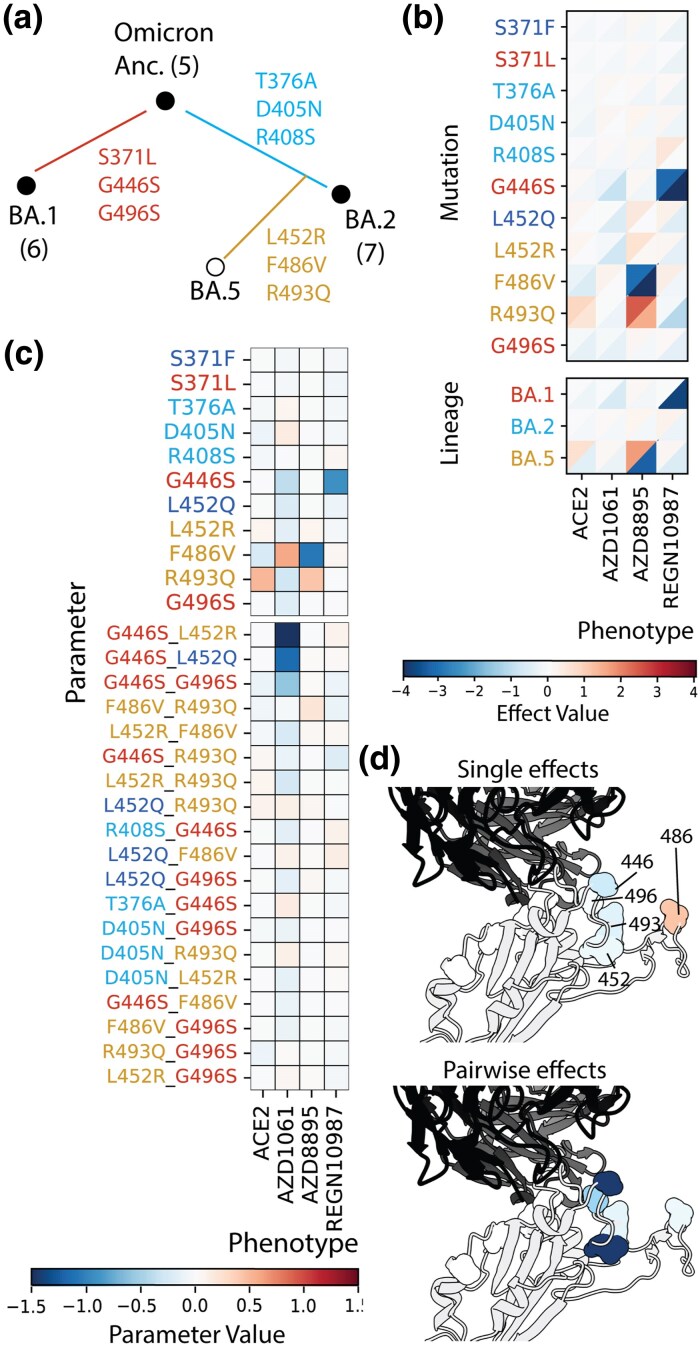
Effects of and interactions between BA.1/BA.2/BA.5 mutations in Library B. a) Evolutionary tree depicting the genotypic relationships among the defined Omicron “ancestor,” BA.1, BA.2, and BA.5 subvariants. Library B mutations are positioned according to the clade on which they first emerged. b) Heatmaps showing the single-mutation effect values (color scale: low → high, blue → red), across all mutational backgrounds for each binding affinity (−log *K*_D,app_) phenotype (*x*-axis). The upper left triangle indicates the 75th percentile of mutation effects, while the lower right triangle represents the 25th percentile. Single-mutation effects are grouped by the viral lineage where the mutation naturally occurs (BA.1, BA.2, and BA.5), with cumulative effect for each background displayed below the single-site effects. Mutation names are colored according to this grouping: red for BA.1 mutations (S371L, G446S, and G496S), light blue for mutations shared between BA.2 and BA.5 (T376A, D405N, and R408S), orange BA.5-specific mutations (L452R, F486V, R493Q), and dark blue for other mutations (S371F and L452Q). c) Heatmaps of inferred model parameters for single and pairwise mutations, evaluated across different phenotypes for models truncated at order 2. Pairwise interactions with a sum of absolute values exceeding 0.25 are shown, where each mutation pair is separated by a dash. Parameter names are colored based on scheme in a). d) Fab AZD1061/Wuhan Hu-1 RBD cocrystal structure (PDB: 7L7E; Dong et al. 2021) with AZD1061 in black and the RBD in white, with surfaces shown for the Library B residues. Residues are colored by the inferred single-mutation or pairwise interaction effects (negative → positive: red → blue) shown in c).

Omicron BA.1, BA.2, and BA.5 each carry lineage-defining mutations (all included in Library B; Fig. 2a), with each lineage affecting distinct sets of measured phenotypes (Fig. 2b). Specifically, BA.1 mutations (especially G446S) confer REGN10987 evasion, whereas BA.5 mutations (F486V and R493Q) affect ACE2 and AZD8895 binding affinities. In contrast, AZD1061 affinity appears to be similarly affected by mutations in both lineages. Interestingly, BA.2 mutations show little contribution to either enhanced ACE2 affinity or evasion from antibodies in our assays. Therefore, in our assays, BA.1 and BA.5 mutations show stronger effects on the measured phenotypes, whereas BA.2 mutations may have smaller effects, potentiate later adaptation, or primarily impact evasion from antibodies not represented here.

In general, Library B variants bind ACE2 with higher affinity and monoclonal antibodies with lower affinity than the Wuhan Hu-1 variant (Fig. S2). This suggests that most combinations of BA.1 and BA.5 mutations do not substantially reduce fitness. Additionally, about one-third of the library binds ACE2 with even higher affinity than BA.1, BA.2, and BA.5 variants, and a large proportion of the library also shows reduced affinity compared with these variants of concerns. Thus, some alternative mutation combinations may actually improve fitness relative to established variants. Indeed, the addition of further mutations on the Omicron ancestral genotype does not decrease the average ACE2 affinity while simultaneously reducing binding to the monoclonal antibodies (Fig. S3). This pattern may result from the accumulation of strongly beneficial mutations or from potential synergistic epistasis between mutations from different lineages. The latter is particularly relevant given the possibility that recombinants between distinct lineages could emerge with higher-than-additive fitness.

Three phenotypes (ACE2, AZD8895, and REGN10987) do not exhibit significant epistasis (Fig. 2c). We used a biophysical epistasis model (see Materials and methods) to infer single, pairwise, and higher-order mutational effects on each binding phenotype. The inferred single-mutation effects agree with the raw mutation effect distributions (Fig. 2b and c). For instance, the two BA.5 mutations F486V and R493Q affect ACE2 and AZD8895 affinities in opposite directions (beneficial versus deleterious). These effects are largely additive, with only a small interaction between the two on AZD8895. By contrast, the single BA.1 mutation G446 is the only significant effect term for REGN10987.

In contrast, mutations from different lineages interact to affect AZD1061 affinity. Although most mutational effects appear small for this phenotype (Fig. 2b), the best-fitting model recovers significant contributions from both single-mutation and pairwise terms. Among single mutations, only F486V significantly increases AZD1061 binding affinity (i.e. is deleterious for evasion), whereas five mutations individually promote evasion. We also detect three significant negative pairwise interactions between G446S and L452R, L452Q, or G496S, two of which are interlineage. The residues involved in significant interactions (446, 452, and 496) cluster in space (∼4 to 16 Å apart), and residue 446 makes direct contacts with both the antibody heavy and light chains (Fig. 2d). The interaction with L452R/Q is consistent with a local perturbation (i.e. steric effects from increased side-chain size and altered electrostatics from changes in charge/polarity (Fujita et al. 2022; Chen et al. 2024)) that could, in turn, modulate the impact of G446S on binding. Additionally, F486 (the strongest single-mutation effect) is far from the antibody binding interface and the cluster of interacting residues, providing a second example in which AZD1061 affinity is likely modulated indirectly rather than through direct antibody contacts. Altogether, the Library B dataset shows that BA.1- and BA.5-specific mutations affect distinct subsets of phenotypes, yet for AZD1061 evasion, mutations from these different lineages can interact synergistically.

### BA.5c mutations have lineage-specific effects (Library C)

After the BA.5 lineage rose to dominance, several convergent mutations (BA.5C mutations) began to accumulate across its subclades, many of which further improve variant fitness within the lineage. Here, we constructed combinatorial Library C by introducing BA.5C mutations not only onto the BA.5 lineage itself but also across several variant backgrounds. Note that, BA.5C mutations include F486V and R493Q, both of which are characteristic of the BA.5 subvariant. Many of the resulting combinatorial variants exhibit reduced affinity to monoclonal antibodies, particularly compared with previously established variants (Fig. S4). Nevertheless, these variants largely retain ACE2 binding, with some even displaying higher affinity than the established variants.

**Figure 3:**
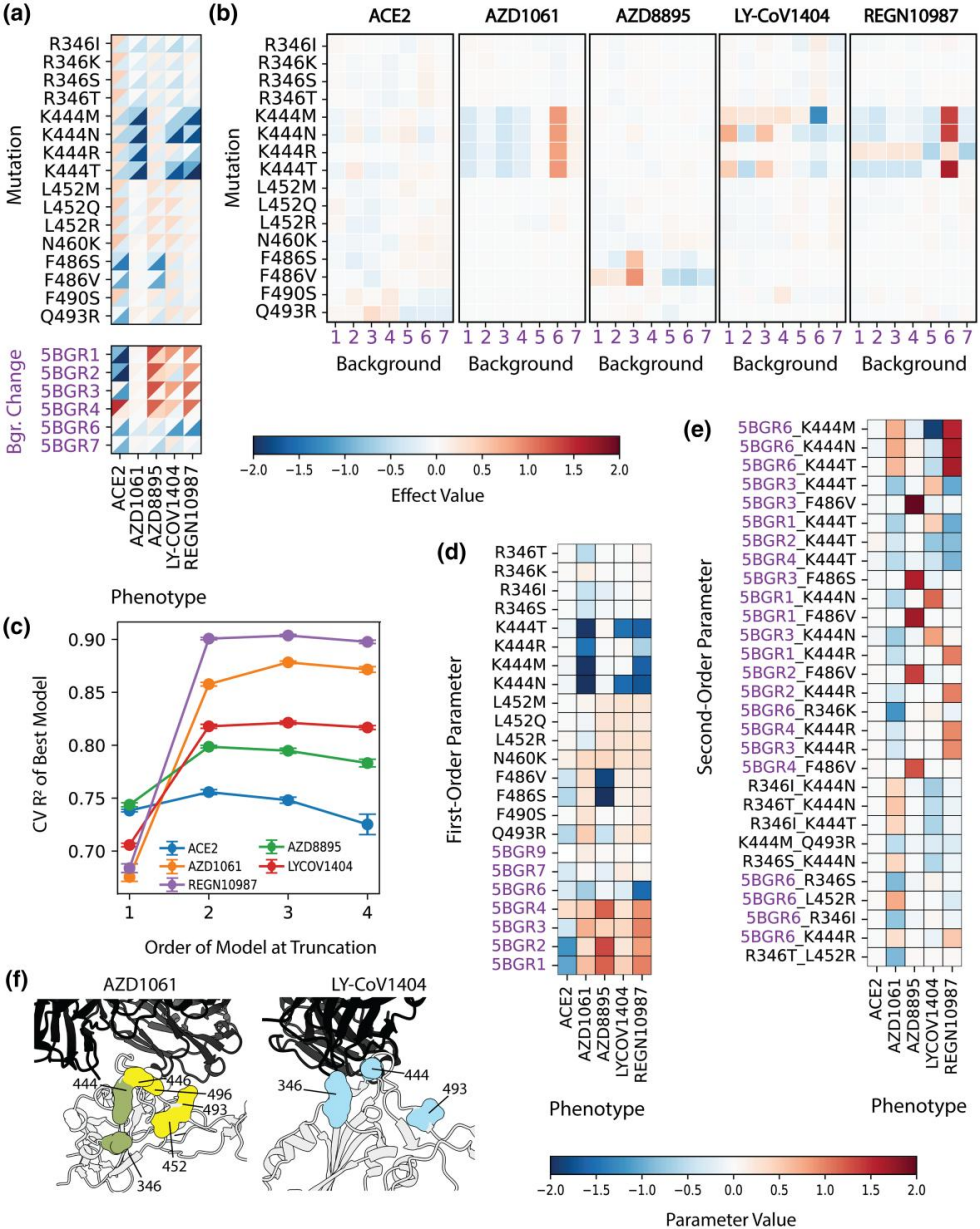
Effects of BA.5C mutations across different genetic backgrounds in Library C. a) Heatmaps showing the single-mutation effect values (color scale: low → high, blue → red) across all mutational backgrounds for each phenotype (*x*-axis). The upper left triangle indicates the 75th percentile, while the lower right triangle represents the 25th percentile of mutational effects. Combined effects of mutations in each background (in purple, relative to background 5, ancestral Omicron) are shown below the single-site effects. b) Heatmaps displaying the mean effect of each mutation (*y*-axis) across different backgrounds (*x*-axis) for each assayed phenotype. To emphasize background-specific effects, the mean across backgrounds is subtracted from each row, effectively mean-centering the data. (c) Cross-validated performance (*R*^2^) for each model across 10-fold cross-validation. Points indicate the mean (*R*^2^) across folds, with error bars representing standard deviation. Heatmaps of inferred model parameters for single d) and pairwise e) mutations, evaluated across different phenotypes for models truncated at order 3. Pairwise interactions with a sum of absolute values exceeding 1 are shown, where each mutation separated by a dash. Background changes are included as one parameter, colored in purple. f) Fab AZD1061/Wuhan Hu-1 RBD (PDB: 7L7E) and Fab LY-CoV1404/Wuhan Hu-1 RBD (PDB: 7MMO; Westendorf et al. 2022) cocrystal structures with Fab antibodies in black and the RBD in white, with surfaces shown for the Library C residues. In the AZD1061 structure, Library B residues exhibiting significant epistasis are highlighted in yellow, and additional interacting Library C residues identified in e) are shown in green. In the LY-CoV1404 structure, Library C residues involved in epistasis for affinity are shown in blue.

Individually, BA.5C mutations have little effect on ACE2 affinity (Fig. 3a). Instead, much of the observed variance in ACE2 binding for Library C can be explained by the underlying genetic backgrounds. Consistent with Library A results, the compensatory mutations Q498R and N501Y (Background 4) improve ACE2 affinity. As seen in Library B, the BA.1- and BA.2-specific mutations (Backgrounds 6 and 7) slightly reduce ACE2 affinity compared with the ancestral Omicron background (Background 5), yet their ACE2 affinities remain higher than that of the Wuhan Hu-1 variant (Background 1). In contrast, lineage-defining BA.5 mutations themselves have relatively weaker effects on ACE2 affinity. Although substitutions at RBD sites 486 and 493 generally reduce binding, these effects are mitigated in BA.5 through two mechanisms: (i) The F486V/S substitutions are less deleterious on Omicron-derived backgrounds (Fig. 3b; Backgrounds 5 to 7) and (ii) the Q493R substitution is largely replaced by its reversion, R493Q. Therefore, among the BA.5C mutations, only the fixed substitutions R493Q and F486V contribute to maintaining ACE2 affinity.

In contrast, BA.5C mutations strongly promote antibody evasion, with substitutions at RBD site K444 being particularly effective against all Class 3 antibodies tested: AZD1061, LY-CoV1404, and REGN10987 (Fig. 3a). However, the effects of K444 and other BA.5C mutations differ across genetic backgrounds (Fig. 3b), as also reflected in the epistasis model predictions and inferred parameters (Fig. 3c to e). For ACE2 affinity, including higher-order interaction terms only marginally improves or even reduces model performance (Fig. 3c), whereas pairwise terms markedly improve predictions for antibody-binding phenotypes. These apparent interactions may reflect true epistasis between mutation and background or limits of measurement sensitivity. For example, only two K444 alleles (T and N) confer escape from LY-CoV1404, while K444M mediates escape only on the BA.1 background (Background 6), indicating indeed an interaction between this site and BA.1 mutations (Fig. 3b and e). In contrast, for AZD1061 and REGN10987, BA.1 mutations themselves already reduce binding relative to the Omicron ancestor (Fig. 3a and d), which likely dampen the apparent impact of K444 mutations on this background (Fig. 3b and e). This dampening is particularly evident for REGN10987, which is effectively escaped by the BA.1 variant, as previously observed (Moulana et al. 2023). Altogether, although BA.1 mutations alone are already effective at evading Class 3 antibodies, particularly REGN10987 and AZD1061, the acquisition of K444 mutation is either sufficient or necessary (as in the case of LY-CoV1404) to achieve broader Class 3 antibody escape.

Although not to the extent of the strong K444 mutations, other BA.5C substitutions still affect antibody escape, sometimes with background dependence. As observed in Library B, F486V/S variants effectively escape the class 1 antibody AZD8895, which is not escaped by K444. These effects are less pronounced in pre-Omicron backgrounds (Backgrounds 1 to 4; Fig. 3b), suggesting that acquisition of F486V/S in BA.5 specifically contributes to AZD8895 or similar antibody escape. R346 mutations, in contrast, contribute to AZD1061 escape with minimal background dependence. R346T interacts with L452R to produce stronger AZD1061 evasion effects (Fig. 3e) and also interacts with K444N/T for enhanced LY-CoV1404 escape. R346, L452, and K444 lie in the same structural neighborhood as residues implicated in Library B (G446, L452, Q493, and G496), consistent with a local cluster of sites whose mutations interact to modulate AZD1061 affinity (Fig. 3f). In contrast, the interaction between R346 and K444 mutations on LY-CoV1404 affinity may be mediated more directly through antibody contacts, since both residues contribute to the binding interface. Despite these specific examples, interactions between two BA.5C mutations are much rarer than those involving a background change (Fig. 3e), indicating that the landscape is largely shaped by background dependence rather than interactions among BA.5C mutations themselves.

While phenotypic landscapes spanned by Library C are largely additive (Fig. 3c to e), the specific compatibility of BA.5C mutations within their native lineage backgrounds (Backgrounds 5 and 7) suggests that this type of interactions may have influenced their evolutionary trajectory. However, this background dependency, along with the diverging effects of individual mutations across different phenotypes, may have constrained BA.5 lineage evolution. Although BA.5 experienced a rapid expansion, its dominance was short-lived, as it was soon displaced by BA.2 recombinants, such as XBB, and later by JN.1. These patterns highlight the dynamic nature of SARS-CoV-2 evolution, where the fitness landscape continuously shifts due to both mutation interactions and immune-driven selection pressures.

### JN.1 and XBB mutations slightly prefer BA.2 lineage (Library M)

**Figure 4:**
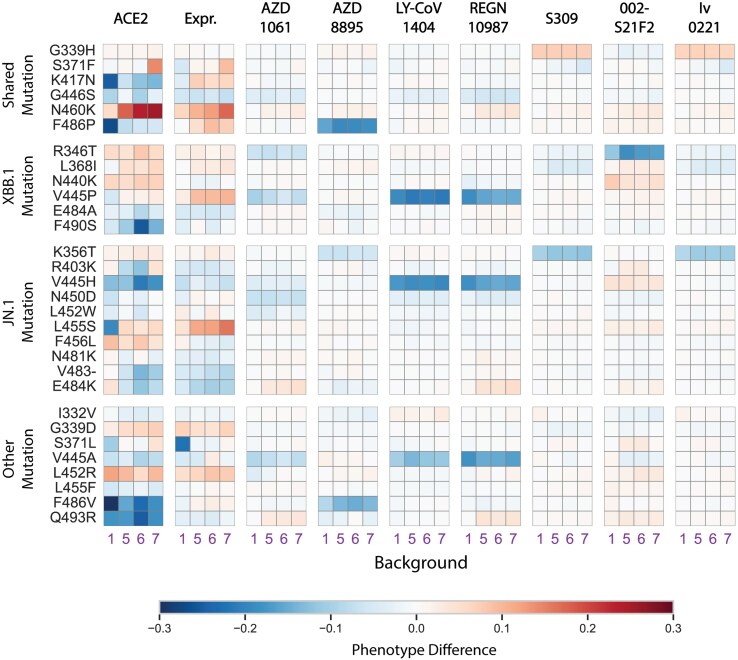
Library M XBB and JN.1 mutation effects across different genetic backgrounds. Heatmaps showing the mean effect of each mutation (*y*-axis) across different genetic backgrounds (*x*-axis) for each measured phenotype. For each background, the effect of a mutation is calculated as the difference between the mean phenotype of genotypes carrying the mutation and the mean phenotype of genotypes lacking it. Mutations are grouped by their occurrence in both lineages, only the XBB lineage, only the JN.1 lineage, or other BA.2 lineages.

The dominance of the BA.5 lineage waned following the reemergence of BA.2-derived recombinant lineages, notably XBB, a recombinant between BA.1 and BA.2, and subsequently JN.1. These lineages harbor distinct yet overlapping sets of prevalent mutations relative to their ancestral BA.2 background (Figs. 1b and c and  4). To investigate the phenotypic impacts of these mutations, we generated variant libraries consisting of random combinations of JN.1 and XBB mutations introduced onto four representative genetic backgrounds (Wuhan Hu-1, ancestral Omicron, BA.1, and BA.2; Table 1). Consequently, our data provide statistical summaries of phenotype effects for each mutation within these distinct genetic backgrounds rather than exhaustive measurements on each possible combinatorial background, as was done for Libraries A, B, and C (see Materials and methods).

We find that the effects of XBB.1 and JN.1 mutations on ACE2 affinity and protein expression depend strongly on genetic background (Fig. 4). Five mutations shared between the two lineages (N460K, S371F, F486P, G446S, and K417N) substantially alter ACE2 binding, but their effects are highly background dependent. N460K and S371F improve ACE2 affinity on Omicron-derived (5 to 7) backgrounds but have little impact on the ancestral Wuhan Hu-1 (1) background. Conversely, F486P, G446S, and K417N are more deleterious on BA.1 (6) and Wuhan Hu-1 (1) backgrounds. Together, these patterns indicate a modest preference for BA.2 (7) and ancestral Omicron (5) backgrounds. Expression levels show a similar trend, with stronger beneficial effects on BA.2 and other Omicron-derived contexts. The same background dependency extends to lineage-specific substitutions: for example, JN.1-specific mutations R403K and L455S enhance ACE2 binding on the BA.2 background despite being deleterious elsewhere. Overall, background-dependent effects dominate the phenotypic variance in ACE2 affinity and are generally stronger than any detectable pairwise mutation interactions (Fig. S5).

In contrast to their effects on ACE2 affinity, the effects of these mutations on antibody evasion are more consistent across genetic backgrounds. Most shared XBB.1 and JN.1 mutations do not confer substantial escape, except for F486P, which reduces binding to AZD8895. Evasion from LY-CoV1404 and REGN10987 arises from distinct substitutions at the same residues in each lineage, suggesting convergent solutions to immune pressure. Notably, the last three antibodies tested show lineage-restricted escape. R346T, unique to XBB.1, strongly reduces binding by 002-S21F2, an antibody known to neutralize both BA.1 and BA.2 (Kumar et al. 2022). Meanwhile, K356T, specific to JN.1, mediates escape from S309 and Iv0221—broadly neutralizing antibodies that remain effective against all prior Omicron variants. The partial evasion of these antibodies by K356T underscores the capacity of JN.1 and its descendants to further erode broad antibody protection. While most evasion phenotypes appear additive, especially with backgrounds, several substitutions exhibit measurable epistasis, sometimes exceeding background effects (Fig. S5). Detecting higher-order interactions, however, will require substantially larger datasets and more precise assays.

Together, these findings suggest that the emergence of the XBB.1 and JN.1 lineages reflects a balance between maintaining ACE2 affinity and broadening immune evasion. Several key escape mutations (e.g. V445P, V445H, and F486P) reduce ACE2 affinity, but these deleterious effects are mitigated by compensatory substitutions such as N460K and S371F, both of which are more beneficial on the Omicron BA.2 background. Similarly, across Libraries C and M, mutation effects tend to be more beneficial or less deleterious on Omicron-derived backgrounds (Figs. 3 and 4). However, among themselves, post-Omicron mutations are largely additive, with little evidence of strong epistasis, particularly for ACE2 affinity. This contrasts with Library A results, where the ACE2 landscape appears more rugged than those of antibody escapes. Consequently, the phenotypic landscapes following initial Omicron diversification seem relatively smooth, with most interactions confined to those between new and ancestral Omicron mutations rather than among the new mutations themselves. This may help explain why adaptive substitutions continued to accumulate within Omicron-derived lineages with little constraint, as the lineage itself appears to potentiate the acquisition of new mutations compared to its Wuhan Hu-1 predecessor.

## Discussion

Throughout its evolutionary history, SARS-CoV-2 has continuously acquired mutations, particularly concentrated within the RBD of its spike protein. The phenotypic effects of these mutations can vary substantially depending on the genetic background at other loci, often resulting in complex interactions (Sailer and Harms 2017; Moulana et al. 2022). Such epistatic interactions can enable certain mutations to compensate for otherwise deleterious effects on distinct phenotypes (Moulana et al. 2022). In this study, we systematically explored this complexity by characterizing multiple genotype spaces across different phenotypic traits. Specifically, we constructed multiple combinatorial libraries reflecting diverse evolutionary branches of the Omicron lineage and measured several binding phenotypes using high-throughput experimental assays.

In the evolutionary history of the Omicron variant, the initial BA.1 variant was rapidly replaced by subsequent BA.2 and BA.5 variants, likely reflecting the higher relative fitness of the BA.2 and BA.5 variants given immune pressures at the time (Bloom and Neher 2023). However, none of the phenotypes assayed here are affected by the BA.2 mutations. Because BA.2 nonetheless displaced BA.1, it likely possessed a fitness advantage arising from selective pressures not captured in our assay panel (e.g. immune pressures outside the specific antibodies measured here). Indeed, BA.1, BA.2, and BA.5 show distinct antibody-escape profiles, suggesting that each subvariant experienced partially different immune pressures during its emergence (Shrestha et al. 2022; Cao et al. 2022a). Notably, BA.5 demonstrates broader antibody evasion compared with BA.1 and BA.2, including resistance to immunity induced by previous infections with these earlier Omicron subvariants (Kimura et al. 2022; Yajima et al. 2024). Despite these differences, we found beneficial mutations unique to each lineage (e.g. BA.1 and BA.5) to be not necessarily incompatible with each other. Therefore, mutations from both lineages may combine and produce variants of even greater concern. More strikingly, we observed that mutations from distinct lineages may even interact synergistically (Fig. 2). For instance, the BA.1 mutation G446S shows beneficial epistasis with BA.5 mutations. Such interactions may have facilitated the emergence and subsequent spread of recombinant variants, particularly those carrying G446S, following the dominance of BA.5 (Carabelli et al. 2023; Garcia et al. 2024).

Within the BA.5 lineage itself, several variants emerged carrying BA.5C mutations that were not initially fixed but later converged across independent evolutionary branches (Ao et al. 2023; Ito et al. 2023). This diversification within BA.5 may have been driven by heterogeneity in population-level immunity (Lugano et al. 2024; Chemaitelly et al. 2025). In our analysis of Library C, which encompassed combinations of some of these mutations, we found that mutation effects were typically independent of genetic background. Nevertheless, in certain cases, specific mutations exerted stronger beneficial effects on the BA.2 (BA.5's progenitor) genetic background (Background 7), such as mutations at position 444, consistent with other neutralization and binding datasets (Starr et al. 2022; Witte et al. 2023). We also recovered potential interactions affecting escape from Class 3 antibodies (AZD1061 and LY-CoV1404) between mutations at K444 and R346. Notably, the combination of K444T, N460K, and R346T defines the BQ.1.1 variant, which rose to prominence in part due to its potent evasion of Class 3 RBD-targeting antibodies. (Qu et al. 2023). Following this pattern, BA.5 was eventually displaced, first by the XBB variant and subsequently by JN.1, potentially reflecting the repetition of the initial Omicron evolutionary dynamics.

The subsequent evolution of post-Omicron lineages was characterized by the accumulation of numerous mutations across different BA.2-derived clades. As a result, unlike in pre-Omicron lineages, many distinct combinations of mutations could give rise to potentially fit variants. One such example is the XBB lineage, a recombinant between two BA.2 descendants, BJ.1 and BM.1.1.1, which rapidly rose to dominance (Tamura et al. 2023). The recombination breakpoint lies within the RBD, where each parental segment contributes escape from distinct antibody profiles (Arora et al. 2023; Tamura et al. 2023). Following the dominance of the XBB clade, the JN.1 lineage emerged, evolving from the older BA.2-derived subvariant BA.2.86 (Kaku et al. 2024). One notable mutation unique to this clade is L455S, which was found to enhance ACE2 affinity (Liu et al. 2024). Interestingly, we found that this effect becomes deleterious on the Wuhan Hu-1 background (Fig. 4). More generally, for ACE2 landscape, interactions between the examined mutations and backgrounds are more prevalent than interactions among the mutations themselves. Conversely, weak intermutation interactions do appear in the antibody-evasion landscapes, but their effect sizes are relatively small compared to those of single mutations (Fig. S5). Thus, the explosion of BA.2 derivatives might not have resulted from significant interaction between these novel mutations but from the interaction with the ancestral mutations (Witte et al. 2023). As a result, different branches could co-occur, and new variants stemming from older clades might emerge, which would have been unlikely on the pre-Omicron clades.

Leveraging our high-throughput phenotype measurements, we have generated detailed genotype-to-phenotype maps for several regions of the SARS-CoV-2 RBD mutational landscape. Specifically, our results highlight scenarios in which viral evolution follows primarily additive landscapes and other cases where it traverses more epistatic spaces. Nonetheless, our current analysis is limited to mutations within the RBD region and thus cannot account for potential epistatic interactions with other protein domains. Additionally, our assays assessed up to 300,000 variants, which, although extensive, still represent only a fraction of the vast combinatorial mutation space. Due to the constraints of our measurement system, our datasets include affinity measurements for only a limited set of antibodies and therefore do not comprehensively capture the breadth of immune-selection pressures. More comprehensive datasets that cover additional mutation combinations, broader phenotypes, and more precise measurements are needed to enhance the predictive accuracy and generalizability of evolutionary models. Moving forward, these methods can be further utilized not only to refine predictive genotype-to-phenotype models but also to provide critical insights into the dynamics underlying viral evolution itself.

## Materials and methods

### Yeast display plasmid, strains, and library production

To generate clonal yeast strains, we used previously generated pETcon-based plasmids expressing Wuhan Hu-1 (pOM102; pAB101), Omicron ancestor (pOM104; pAB105), and Omicron BA.1 (pOM101; pAB106) RBDs. In addition to the RBD coding sequence, which is fused to Aga2 and tagged with myc, this plasmid also includes ampicillin resistance cassette for bacterial selection and a yeast mating factor for protein localization. Using site-directed mutagenesis (NEB #E0554S), we generated additional plasmids expressing RBD variants Wuhan Hu-1 + Q498R (pAB102), Wuhan Hu-1 + N501Y (pAB103), and Wuhan Hu-1 + Q498R + N501Y (pAB104). We also produced an Omicron BA.2 construct via Gibson assembly, replacing the RBD sequence with a gBlock (IDT DNA). All plasmids were amplified in NEB 10-Beta cells and extracted from saturated cultures (Geneaid #PD300). We then transformed Sanger-verified plasmids into the AWY101 yeast strain (Wentz and Shusta 2007) as described in Gietz and Schiestl (2007).

To construct RBD variant libraries, we used a Golden Gate combinatorial assembly strategy. Full-length RBD sequences were assembled from sets of dsDNA fragments of roughly equal size, with each set containing fragment versions that differed by the mutations included. For Library B and Library C, fragments were amplified from corresponding plasmids (pAB104 for Library B; pAB101 to pAB107 for Library C) using primers introducing the desired mutations and flanked by BsaI recognition sites (File S1). For Library M, we produced four fragments: the first two incorporated all combinations of mutations shown in Fig. 1a, while the last two included randomly selected combinatorial mutations (Python randomization). We ordered oligo pools (TWIST Bioscience) containing the additional BsaI sites and distinct flanking sequences for each fragment and genetic background. Separate Golden Gate assembly reactions were performed for each library, generating between 2.4 and 10 million *Escherichia coli* colonies after transformation and recovery in 100 mL of molten lysogeny broth (LB; 1% tryptone, 0.5% yeast extract, and 1% NaCl) containing 0.3% SeaPrep agarose (VWR, Radnor, PA, USA #12001-922) with ampicillin, spread into a thin layer (about 1 cm deep). We then extracted plasmid DNA from the pooled colonies, transformed the libraries into the AWY100 yeast strain, and recovered them in 100 mL of molten synthetic dextrose and CAS amino acids (SDCAA; 6.71 g/L YNB without amino acids [Sigma-Aldrich #Y0626], 2% dextrose [VWR #90000-904], 5 g/L Bacto casamino acids [VWR #223050]), containing 0.5% SeaPrep agarose. From these, we inoculated in liquid SDCAA and froze libraries containing ∼1 million yeast colonies each.

### High-throughput binding affinity assays (Tite-Seq, Sort-Seq, and enrichment)

For some measurements (see Table 1), we performed Tite-Seq assays as previously described (Adams et al. 2016; Starr et al. 2020; Phillips et al. 2021; Moulana et al. 2022), with two replicates for each antibody or ACE2. Briefly, we thawed a given yeast RBD library by inoculating the corresponding glycerol stocks in SDCAA (6.7 g/L YNB without amino acid [VWR #90004-150], 5 g/L ammonium sulfate [Sigma-Aldrich #A4418], 2% dextrose [VWR #90000–904], 5 g/L Bacto casamino acids [VWR #223050], 1.065 g/L 2-(N-morpholino)ethanesulfonic acid (MES) buffer [Cayman Chemical, Ann Arbor, MI, USA, #70310], and 100 g/L ampicillin [VWR #V0339]) at 30 °C for 20 h. The cultures were then induced in SGDCAA (6.7 g/L YNB without amino acid [VWR #90004-150], 5 g/L ammonium sulfate [Sigma-Aldrich #A4418], 2% galactose [Sigma-Aldrich #G0625], 0.1% dextrose [VWR #90000–904], 5 g/L Bacto casamino acids [VWR #223050], 1.065 g/L MES buffer [Cayman Chemical #70310], 100 g/L ampicillin [VWR #V0339]), and rotated at room temperature for 18 h.

Following overnight induction, we pelleted, washed (with 0.01% phosphate buffer saline with bovine serum albumin [PBSA; VWR #45001-130; GoldBio, St. Louis, MO, USA, #A-420-50]), and incubated the cultures with monoclonal antibody or ACE2 at corresponding concentrations. The yeast–antibody mixtures were incubated at room temperature for 20 h. The cultures were then pelleted washed twice with PBSA and subsequently labeled with phycoerythrin (PE)-conjugated goat antihuman IgG (1:100, Jackson ImmunoResearch Labs #109-115-098; for antibodies) or PE-conjugated streptavidin (1:100, Thermo Fisher #12-4317; for RBD) and FITC-conjugated chicken anti-cMyc (1:100, Immunology Consultants Laboratory Inc., Portland, OR, USA, #CMYC-45F). The mixtures were rotated at 4 °C for 40 min and then washed twice in 0.01% PBSA.

After incubation and washing, we sorted cells on a BD FACS Aria Illu. Cells were gated by forward scatter (FSC) vs side scatter (SSC) and then by expression (fluorescein isothiocyanate; FITC) and/or binding fluorescence (PE). The machine was equipped with 405, 440, 488, 561, and 635 nm lasers and an 85 micron fixed nozzle. Note that, prior to further quantitative assays, we enriched Library M for ACE2 binders (PE+ population) by sorting, recovering, and freezing 8 million ACE2 binding cells. This sorted population is then used for subsequent assays for Library M.

In the “Tite-Seq and Sort-Seq” assays, FITC gates were first drawn, and FITC+ populations are sorted into four PE (binding) gates, where the first gate is PE− (nonbinding) subpopulation, and PE2, PE3, and PE4 are three PE+ gates with equal population size. Both Tite-Seq and Sort-Seq assays are conducted very similarly, where Tite-Seq assay uses a titration framework with many concentrations (10^−12^ to 10^−6^ M at 0.5 log interval) and Sort-Seq assay on a few concentrations (two concentrations for Library C antibody assay, 10^−6^ and 10^−8^ M, or one concentration for Library M ACE2 assay, 10^−7^ M). In particular, Sort-Seq allows us to maximize the number of antibodies used (Library C) and the number of cells sorted (Library M). In contrast, “enrichment” assays for antibodies on Library M sort cells into one, instead of four or more, populations. Here, a single PE/FITC gate is drawn, where either nonbinding or binding cells are collected, whichever is the smaller population.

In total, for each sample (i.e. independent concentration) across gates, we sorted ∼200,000 yeast cells from Library B, ∼1 million yeast cells from Library C, and ∼4 to 9 million yeast cells from Library M. Sorted cells were then pelleted, resuspended in SDCAA, and rotated at 30 °C until late-log phase (OD600 = 0.9 to 1.4). The cultures were then pelleted and stored at −20 °C for at least 12 h prior to extraction using Zymo Yeast Plasmid Miniprep II (Zymo Research #D2004), following the manufacturer's protocol. The sequencing amplicon libraries were then prepared by a two-step PCR as previously described (Moulana et al. 2022; Phillips et al. 2021; Nguyen Ba et al. 2019).

In brief, we added to the amplicon unique molecular identifiers (UMIs), inline indices, and partial Illumina adapters through a seven-cycle PCR which amplifies the RBD sequence in the plasmid (File S2). We then used the cleaned product (using 0.9× Aline beads) from the first PCR in the second PCR to append Illumina i5 and i7 indices accordingly. The products were then cleaned using 0.85× Aline beads, verified using 1% agarose gel, quantified on SpectraMax i3, pooled, and verified on TapeStation 5000HS and 1000HS. Final library was quantitated by Qubit fluorometer and sequenced on Illumina NovaSeq SP, supplemented with 10% PhiX.

### Sequence data processing

Following Moulana et al. (2022, 2023), we processed raw demultiplexed sequencing reads to identify and extract the indexes and mutational sites. Briefly, for each antibody, we parse through all FASTQ files and group the reads according to inline indices, UMIs, and sequence reads. We converted accepted sequences into corresponding nucleotide sequences using regular expression with some amount of mismatch tolerance throughout the RBD sequence. Reads containing errors at known mutation sites were removed. Then for each recovered sequence, counts in each sample were estimated by number of UMIs from all samples into a single table.

### Enrichment inference

For enrichment assays, we calculated an enrichment score. For each variant, we estimated the proportion of cells in the sorted population relative to the total population, $p_{s,f}$, as follows:


$$p_{s,f}=\frac{\frac{R_{s,f}}{\sum_{s}\;R_{s,f}}C_{f}}{\frac{R_{s,t}}{\sum_{s}\;R_{s,t}}C_{t}}$$

where $R_{s,f}$ is the number of sequencing reads corresponding to genotype *s* in the fluorescently sorted population and $R_{s,t}$ is the number of reads for the same genotype in the total population. $C_{f}$ and $C_{t}$ denote the number of cells in the fluorescently sorted and total populations, respectively. The enrichment score for variant *s*, $E_{s}$, was then defined as $E_{s}\;=\;p_{s,f}$ if the sorted population (whichever was smaller in the FACS experiment) represented the bound phenotype, and $E_{s}\;=\;1-p_{s,f}$ otherwise. Standard errors were estimated from replicate experiments.

### Variant-specific fluorescence inference

For Sort-Seq and Tite-Seq experiments, using sequencing and flow cytometry data, we calculated the mean log fluorescence of each genotype *s* at each concentration *c*, as follows:


$${\bar{F}}_{s,c}\;=\;\underset{b}{\sum }\;F_{b,c}\;p_{b,s|c},$$

where $F_{b,c}$ is the mean log fluorescence of bin *b* at concentration *c*, and $\;p_{b,s|c}$ is the inferred proportion of cells from genotype *s* that are sorted into bin *b* at concentration *c*, which is estimated from the read counts as:


$$p_{b,s|c}=\frac{\frac{R_{b,s,c}}{\sum_{s}\;R_{b,s,c}}C_{b,c}}{\sum_{b}\;\left(\frac{R_{b,s,c}}{\sum_{s}\;R_{b,s,c}}C_{b,c}\right)}.$$

Here, $R_{b,s,c}$ represents the number of reads from genotype *s* that are found in bin *b* at concentration *c*, and $C_{b,c}$ refers to the number of cells sorted into bin *b* at concentration *c*. This log fluorescence is also used as phenotype values for Sort-Seq measurements.

We then computed the uncertainty for the mean log fluorescence:


$$\delta {\bar{F}}_{s,c}\;=\sqrt{\underset{b}{\sum }\;(\;\delta F_{b,c}^{2}\;p_{b,s|c}^{2}+\;F_{b,c}^{2}\delta p_{b,s|c}^{2})},$$

where $\delta F_{b,c}$ is the spread of the log fluorescence of cells sorted into bin *b* at concentration *c*. The error in $p_{b,s|c}$ emerges from the sampling error, which can be approximated as a Poisson process, such that:


$$\delta p_{b,s|c}=\frac{\;p_{b,s|c}}{\sqrt{R_{b,s,c}}}.$$

### Binding affinity, *K*_D_ inference

For affinity measurements across varying ligand concentrations, we estimated the apparent dissociation constant *K*_D,app_ or each genotype as previously described. Briefly, we fit the logarithmic form of the Hill function to the mean log fluorescence $\;{\bar{F}}_{s,c}$, which we had previously computed, as a function of ligand concentration *c*:


$${\bar{F}}_{s,c}=\mathrm{lo}g_{10}\left(\frac{c}{c+K_{D,s}}A_{s}+B_{s}\right),$$

where $A_{s}$ represents the increase in fluorescence at ligand saturation and $B_{s}$ denotes the background fluorescence level. The fits were performed using the *curve_fit* function in the Python package *scipy.optimize*. Across all genotypes, we imposed bounds on the values of $A_{s}$ to be 10^2^ to 10^6^, $B_{s}$ to be 1 to 10^5^, and *K*_D,s_ to be 10^−14^ to 10^−5^. For each measurement, the inferred *K*_D,s_ values were averaged across two experimental replicates after excluding fits with poor quality ($R^{2}<0.8$ or standard error >1).

### Background effect comparisons

We performed background comparison analyses to assess how mutation effects varied across different genetic backgrounds (Fig. 1c). For Library C, we calculated, for each mutation-background pair (*m*, *g*), the effect of adding mutation *m* across all combinations of other mutations (excluding *m*), with the background *g* treated as part of the combination. For Library M, which was not combinatorially complete, we instead estimated the effect of mutation *m* in background *g* by first computing the average phenotype of genotypes containing *m* and comparing it to the average phenotype of genotypes lacking *m*. The difference between these averages provided the estimated effect of mutation *m* in that background.

### Epistasis analysis

To quantify epistatic interactions in the Library B and Library C datasets, we used the Python package pymochi (Faure and Lehner 2024). Briefly, each inference consists of two concurrent modeling steps. First, an additive model comprises the weights of individual mutations and their interactions. This additive model is then passed to a neural network with two hidden layers to capture nonlinear relationships between the additive biochemical model and the observed fluorescence measurements.

For Tite-Seq measurements, the additive biochemical model corresponds to a simple linear combination of mutation effects (mochi transformation: “Linear”). Specifically, we assume that the logarithm of the apparent dissociation constant, $\log\;K_{D,\mathrm{app}}$, is proportional to the change in free energy. In contrast, for Sort-Seq measurements on Library C, we used the “ThreeFractionBound” transformation for each of the two tested concentrations (see Github repository). In this case, the same additive weights are jointly inferred across both low and high concentration phenotypes, as well as for the expression phenotype. For both Tite-Seq and Sort-Seq measurements, we reported the inferred additive model weights, including first- and higher-order coefficients. The full *K*-order additive biochemical model can be expressed as:


$$\mathrm{pheno}=\;\beta_{0}+\overset{K}{\underset{i=1}{\sum }}\;\underset{m\in M_{i}}{\sum }\;\beta_{m}x_{m,s}$$

where $\beta_{m}$ denotes the coefficient for the combination of mutation *m* (a single-mutation coefficient for i=1 or interaction coefficient otherwise), containing all combinations of *i* mutations.

To determine the optimal value of *K*, we performed 10-fold cross-validation for all *K* ≤ 6. For each *K*, the data were divided into ten subsets, with each serving once as a test set for a model trained on the remaining nine. The optimal *K* was chosen as the value that maximized the average prediction performance (*R*^2^) averaged across the ten test sets.

### Statistical analyses and visualization

All data processing and statistical analyses were performed using R (R Core Team 2025) and Python (Van Rossum and Drake 2009). All figures were generated using ggplot2 (Wickham 2016) and matplotlib (Hunter 2007).

## Supplementary Material

msag100_Supplementary_Data

## Data Availability

Raw sequencing reads have been deposited in the NCBI BioProject database under accession number PRJNA1433816. All associated codes and metadata are available at https://github.com/desai-lab/background_dependence_SARS-CoV-2.
