# Supplementary material for: Epistasis and background dependence in the evolution of Omicron variants of the SARS-CoV-2 spike protein
Source: Mol Biol Evol. 2026 May 16;43(5):msag100. doi: 10.1093/molbev/msag100 (PMC13179739; doi:10.1093/molbev/msag100)
Supplement: msag100_Supplementary_Data [file msag100_supplementary_data.zip › Supplemenatry_Figures.pdf]

SUPPLEMENTARY FIGURES

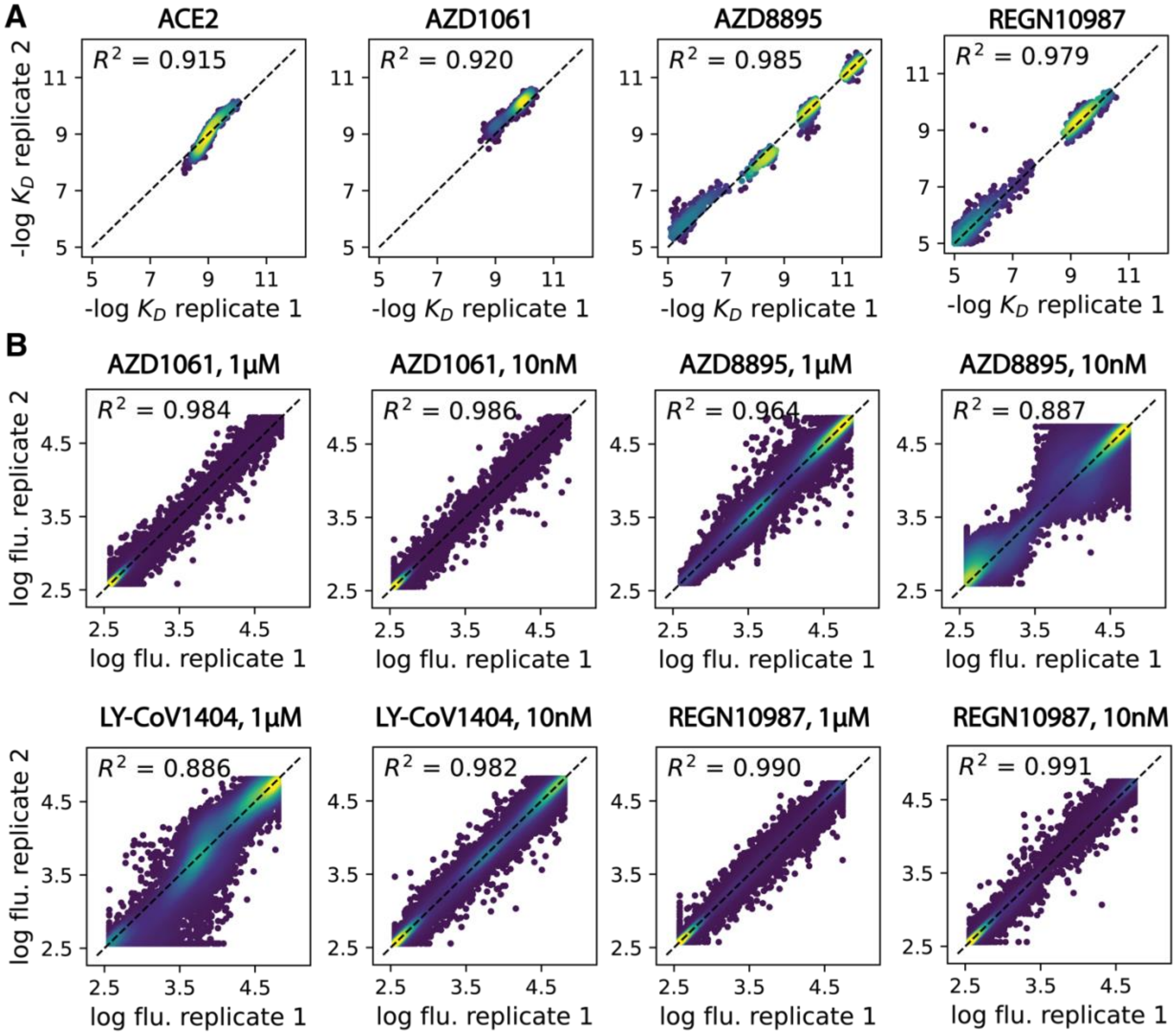

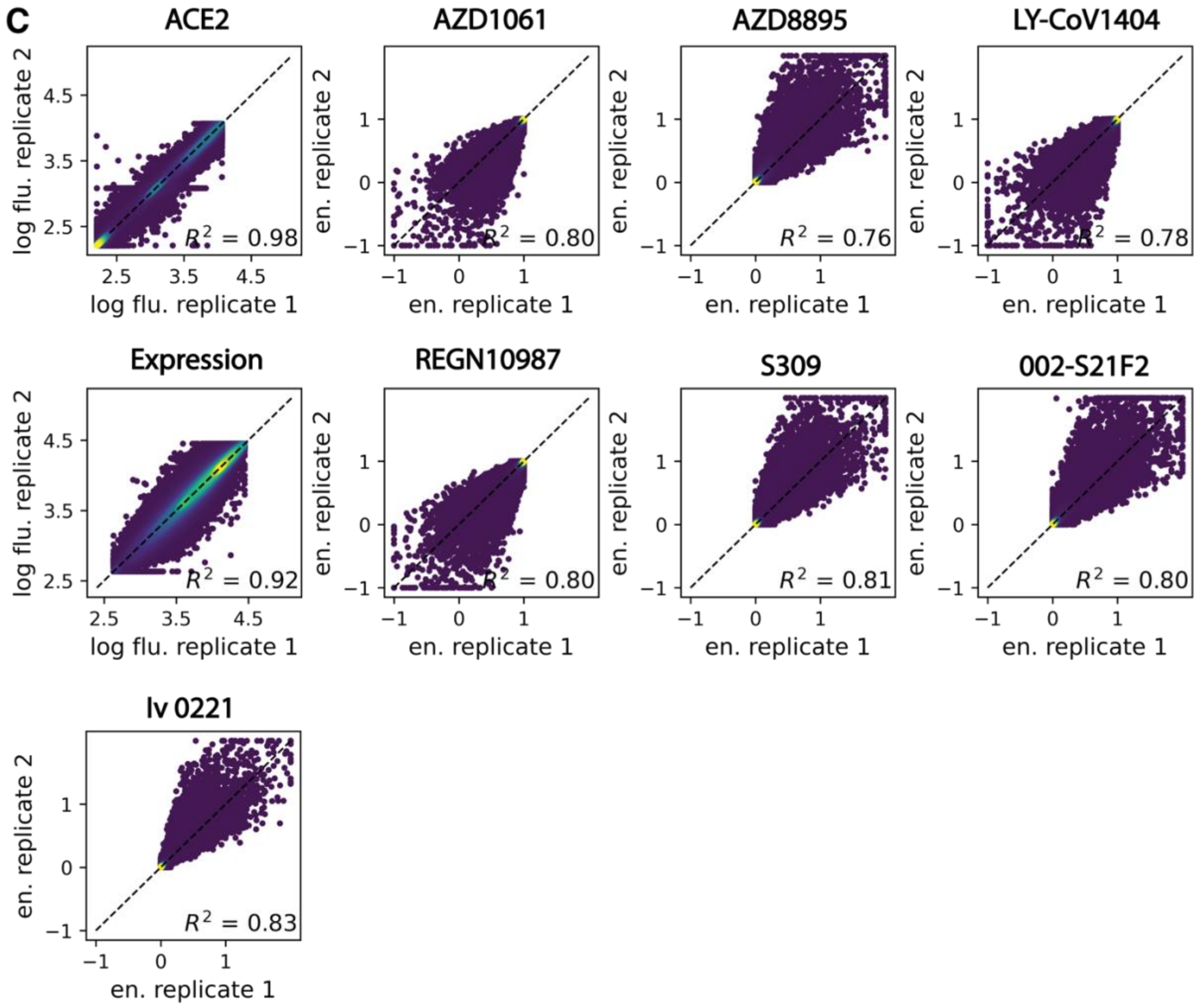

Supplementary Figure 1: Phenotype correlations between measurement replicates. Scatter plots showing replicate correlations for each phenotype measured in Library B (**A**), C (**B**), and M (**C**). Spearman correlation coefficient (R^2) is shown within each plot.

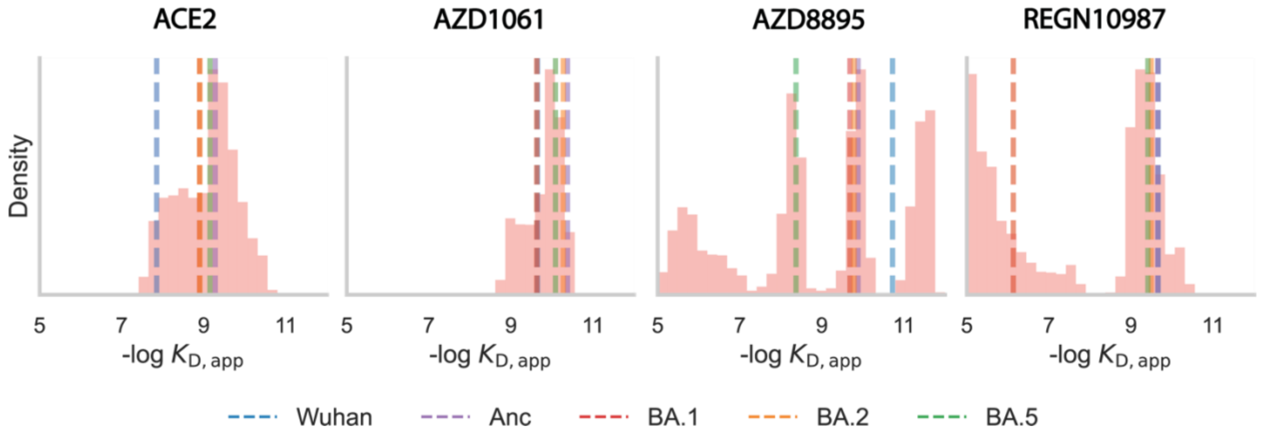

Supplementary Figure 2: Phenotype distribution for Library B variants. Histogram of -log $K_{D,app}$ values for variants in the library. Dashed vertical lines, colored according to the variant, indicate values for specific variants of concern.

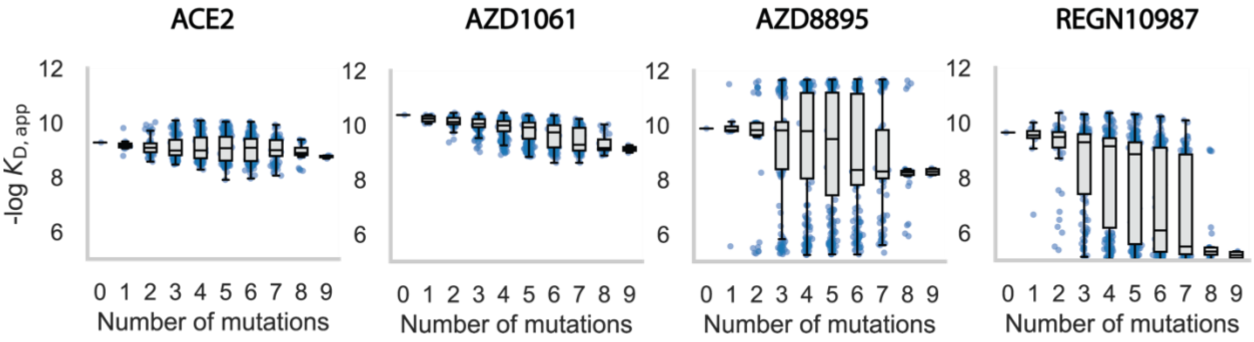

Supplementary Figure 3: Phenotype distribution for Library B variants grouped by the number of mutations. Boxplots of -log $K_{D,app}$ values for variants carrying 0 to 9 mutations for each measured phenotype. In each boxplot, the box represents the interquartile range (25th to 75th percentile), the horizontal line inside the box indicates the median, whiskers extend to the most extreme points within 1.5× the interquartile range, and individual outliers are shown as points with jitter.

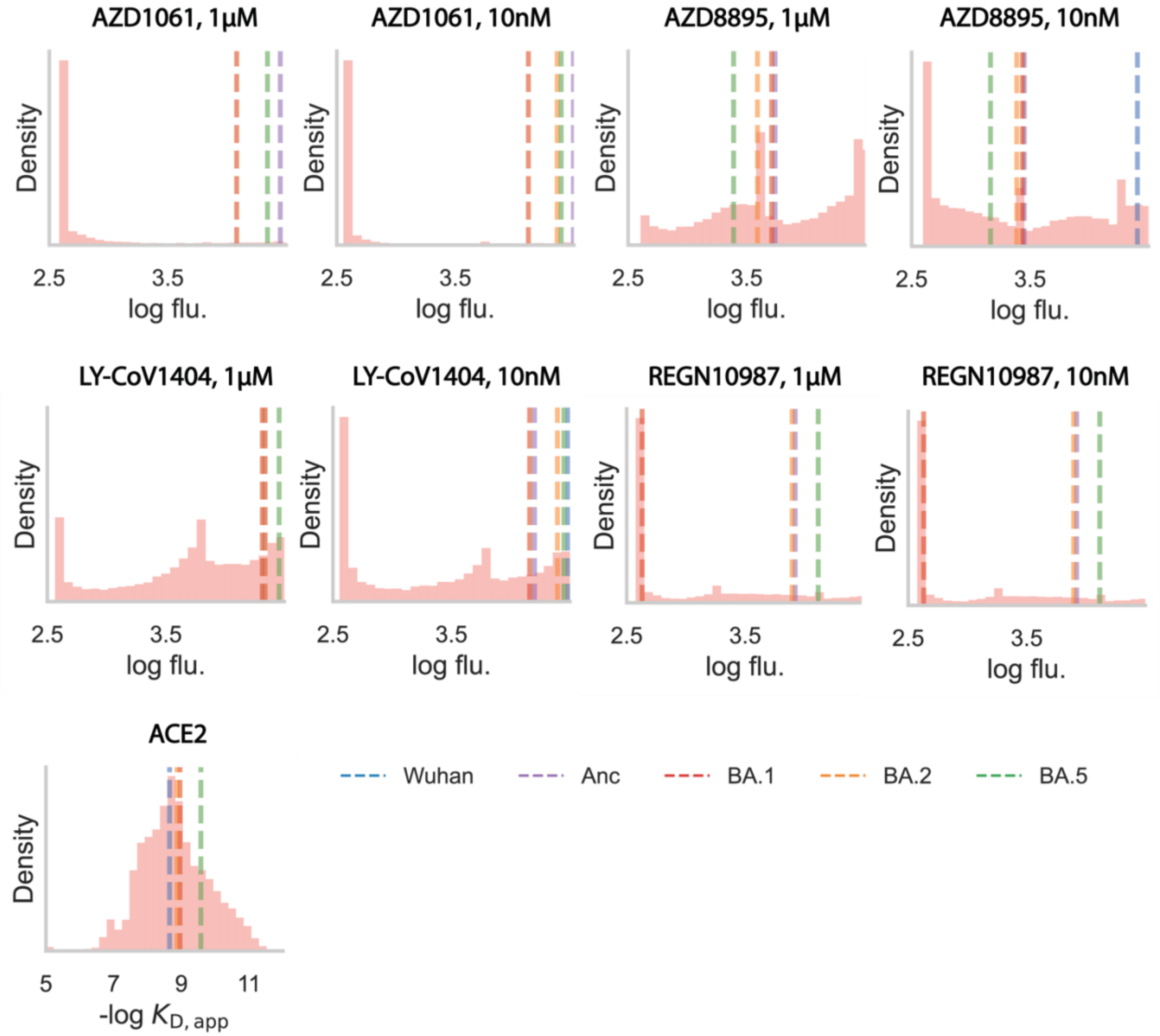

Supplementary Figure 4: Phenotype distribution for Library C variants. Histogram of -log $K_{D,app}$ (for ACE2) and log fluorescence (for mAbs) values for variants in the library. Dashed vertical lines, colored according to the variant, indicate values for specific variants of concern.

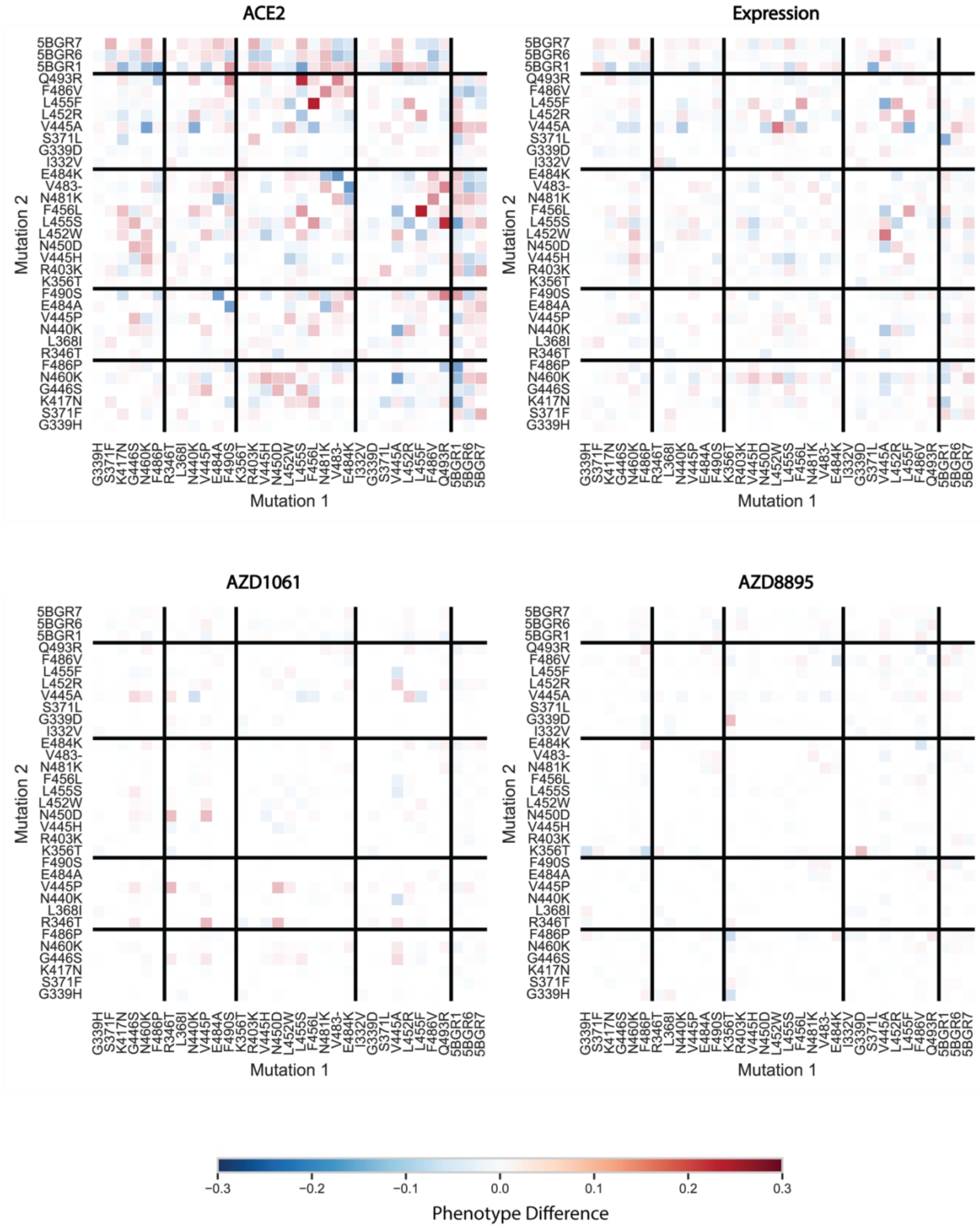
ACE2
Expression
AZD1061
AZD8895
Mutation 1
Mutation 2
−0.3
−0.2
−0.1
0.0
0.1
0.2
0.3
Phenotype Difference

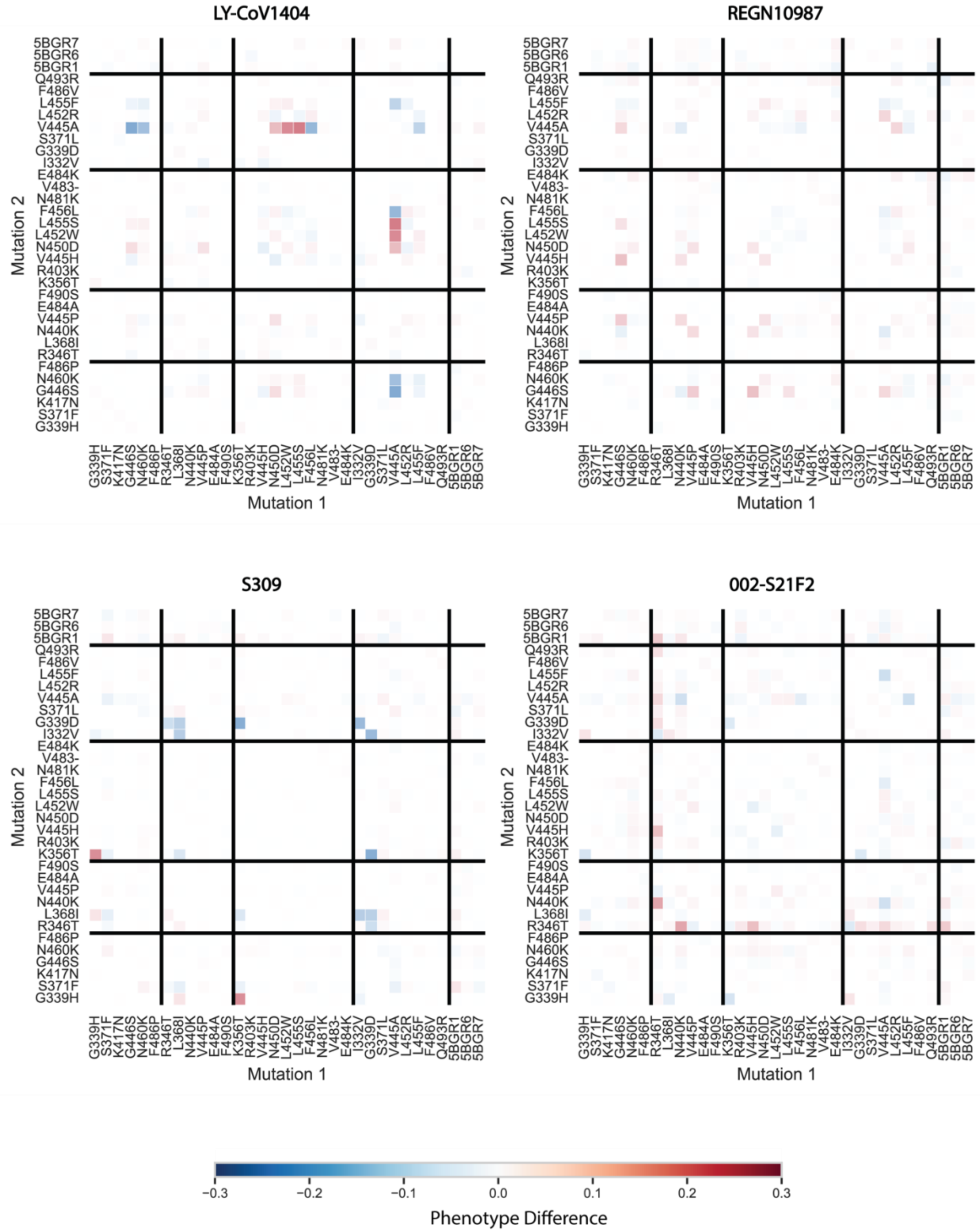

LY-CoV1404
REGN10987
S309
002-S21F2
Mutation 2
Mutation 1
5BGR7
5BGR6
5BGR1
Q493R
F486V
L455F
L452R
V445A
S371L
G339D
I332V
E484K
V483-
N481K
F456L
L455S
L452W
N450D
V445H
R403K
K356T
F490S
E484A
V445P
N440K
L368I
R346T
F486P
N460K
G446S
K417N
S371F
G339H
−0.3
−0.2
−0.1
0.0
0.1
0.2
0.3
Phenotype Difference

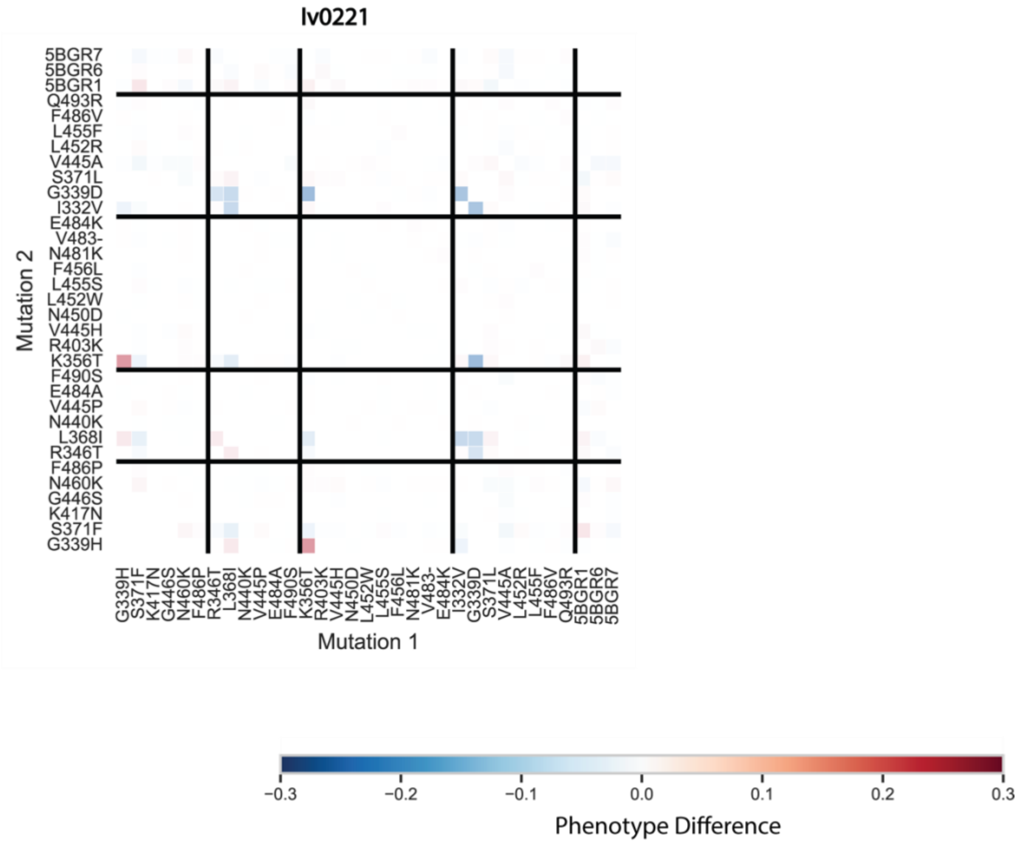

Supplementary Figure 5: Library M mutation/background interactions across measured phenotypes. For each phenotype, heatmap (low → high, blue → red) showing pairwise interaction effects between mutations or backgrounds (x and y axes). Thick black lines separate mutation groups, ordered from bottom to top and from left to right, as follows: shared between XBB and JN.1 lineages, specific to XBB.1 lineage, specific to JN.1 lineage, single mutations not in either lineage, and background changes relative to Background 5. Interactions are calculated as the difference between Mutation 2 effects (each computed as in Figure 4) on genotypes carrying Mutation 1 and on genotypes lacking Mutation 1.
